# Comment on Vicente, F.; Pereira, P.C. Pork Meat Composition and Health: A Review of the Evidence. *Foods* 2024, *13*, 1905

**DOI:** 10.3390/foods13193043

**Published:** 2024-09-25

**Authors:** William B. Grant

**Affiliations:** Sunlight, Nutrition, and Health Research Center, 1745 Pacific Ave., Ste. 504, San Francisco, CA 94109, USA; wbgrant@infionline.net

**Keywords:** Alzheimer’s disease, cognitive performance, pork meat, processed meat, red meat, rheumatoid arthritis, stroke, sustainable diet, type 2 diabetes mellitus

In their recent review, Vicente and Pereira concluded that pork meat can be an option for a healthful and sustainable diet [1]. However, the review not only overlooked many important adverse effects of eating red and processed meat but also overestimated its effect on sustainability.

A scoping review of pork consumption and its relationship to human nutrition and health was published in 2021 [2]. It used data from 86 studies, including 16 randomized controlled trials, 5 other trials, 7 cohort studies, 4 case-cohort and nested case-control studies, 33 case-control studies, and 21 cross-sectional studies. Up to 39 studies were on cancer incidence, 7 were on cardiovascular disease risk factors, and 3 were on risk factors for type 2 diabetes mellitus (T2DM). The authors noted that few conclusions can be drawn from studies on how pork affects human nutrition and health. One reason for this is that most studies were of a short duration of up to 6 months.

In contrast to the statements in Vicente and Pereira [1], the abstract of a 2022 review, also in *Foods* [3], stated the following: “The role of red meat in relation to key physiological conditions such as maintaining skeletal muscle and bone health and during pregnancy is also discussed. The inclusion of lean red meat in a healthy, varied diet may be beneficial during these critical conditions. There is, however, increasing evidence that red meat and especially processed meat are associated with increased risks of [cardiovascular disease], cancer, and dementia, whereas white meat is neutral or associated with a lower risk”.

In their review, Vicente and Pereira claimed on the basis of a meta-analysis of randomized controlled trials that red meat does not reduce risk of T2DM (Ref. 77 in [1]). That analysis reported that, in the trials, red meat consumption did not affect most of the glycemic and insulinemic risk factors for T2DM. However, an analysis of the data from a cohort study involving 162,667 female and 44,975 male healthcare workers monitored for up to 26 years, who completed food frequency questionnaires every four years, showed a strong link between eating meat and the risk of T2DM [4]. The following two empirical dietary patterns showed significant inverse correlations with T2DM incidence: the reversed empirical dietary index for hyperinsulinemia and reversed empirical dietary inflammatory pattern. The hazard ratios for the incidence of T2DM for the 90th vs. 10th percentile, adjusted for many factors such as socioeconomic status and body mass index, for those two dietary patterns were 0.57 (95% confidence interval [CI], 0.57–0.59) and 0.57 (95% CI, 0.55–0.59), respectively. The food groups associated with the strongest risk for chronic diseases in those food groups were red meat and processed meat, along with french fries and energy drinks.

Although Vicente and Pereira also claimed that pork does not negatively affect cognitive performance (Ref. 76 in [1]), I found no such assertion in the cited source.

Alzheimer’s disease (AD) does affect cognitive performance. My research and investigations in the journal literature have led to the conclusion that diet is the most important modifiable risk factor for AD and that meat is the most important contributor to diet-associated risk [5]. I have conducted many single- and multi-country ecological studies regarding AD prevalence with respect to the national dietary supply, e.g., [6]. Such ecological studies have several advantages, including that they enroll a great many participants and that the dietary impact extends to about 20 years. In the reanalysis of my 1997 study (Ref. 8 in [5]), the correlation between meat supply and AD prevalence in 11 countries was *r* = 0.93, *p* < 0.001 (Table 2 in [5]). For this commentary, I analyzed the 11 countries by using pork and nonpork meat supply for 1984–1986 [6]. During that period, the pork dietary supply in those countries ranged from 1.1 to 102 g/day (median, 76 g/day), whereas nonpork meat ranged from 9 to 225 g/day (median, 112 g/day). For nonpork meat, *r* = 0.85, *p* < 0.001, and the Shapiro–Wilk normality test failed, whereas for pork meat, *r* = 0.75, *p* = 0.008. Thus, pork and nonpork meat both contribute to AD prevalence.

A 2007 article cited in Ref. 77 in [1] reported that, in a randomized crossover study in postmenopausal women, the soy–nut regimen decreased low-density lipoprotein cholesterol more than the soy–protein period (−5.0 ± 0.6%; *p* < 0.01) and the control diet (−9.5 ± 0.6%; *p* < 0.01) did [7]. The control diet differed from the soy diets with respect to red meat but not poultry and fish.

Another adverse health effect of eating meat is an increased risk of the incidence and exacerbation of rheumatoid arthritis (RA) [8]. In an ecological study reported in 2000 (Ref. 7 in [8]), RA exacerbations increased in concert with rapidly increasing meat consumption in several European countries around 1970. Fatty acids in meat were the suggested cause. Several articles discussed in [8] offer evidence that eating meat is causally linked to RA risk.

A July 2024 article presented evidence that red meat is also an important risk factor for stroke [9]. In terms of their contribution to global stroke years lived with disability in 2019, red meat (8.1%) was second to high-sodium diets (11.5%). The high-sociodemographic index (SDI) regions were most affected by red meat, whereas high-sodium diets were a major concern in middle-SDI regions.

Vicente and Pereira [1] also did not adequately address the role of pork or red/processed meat in a sustainable diet. The EAT–Lancet Commission on healthy reference diets from sustainable food systems [10] recommended a daily intake of 7 g of pork, 7 g of beef and lamb, and 29 g of poultry. Those values are far below the mean values in most of the world’s developed countries in the period 1984–1986 [6]. Because separating pork meat’s health effects from those of beef and lamb meat is challenging, the effects should be assumed to be similar. Thus, eating pork apparently has many adverse health effects, especially in mid- and late life.

## Data Availability

The original contributions presented in the study are included in the article. Further inquiries can be directed to the corresponding author.
